# Medial Pivot Designs Versus Conventional Bearing Types in Primary Total Knee Arthroplasty: A Systematic Review and Meta-Analysis of Randomized Controlled Trials

**DOI:** 10.5435/JAAOSGlobal-D-22-00170

**Published:** 2022-12-05

**Authors:** Wayne Hoskins, Guy Smith, Tim Spelman, Kelly G. Vince

**Affiliations:** From the Faculty of Medicine, Dentistry and Health Sciences, The University of Melbourne, Parkville, Australia (Dr. Hoskins); Traumaplasty Melbourne, East Melbourne, Australia (Dr. Hoskins); the Department of Orthopaedics, Northland District Health Board, Whangarei, New Zealand (Dr. Hoskins, Smith, and Dr. Vince); and Department of Surgery, St. Vincent's Hospital, the University of Melbourne, Melbourne, Australia (Dr. Spelman).

## Abstract

Medial pivot (MP) designs are growing in popularity. They provide increased sagittal plane stability and theoretically replicate some aspects of native joint kinematics, which may improve total knee arthroplasty outcomes.

**Methods:**

A systematic review was performed of randomized controlled trials (RCTs) that compared MP designs with cruciate-retaining, posterior-stabilized (PS), ultracongruent, or mobile-bearings in primary total knee arthroplasty, according to Preferred Reporting Items for Systematic Reviews and Meta-Analyses guidelines. The primary outcome measures were all clinical function scores, patient-reported outcome measures, and range of motion. The secondary outcome was complications. Two authors independently selected studies, performed data extraction, and risk-of-bias assessment. Studies at high risk of bias were excluded from meta-analysis. Treatment effects were assessed using random-effects meta-analysis and quantified using pooled mean differences or incidence rate differences as appropriate.

**Results:**

Eight RCTs met inclusion criteria. Five compared MP with PS, two with ultracongruent, and one with cruciate-retaining and mobile-bearing. In total, 350 knees were randomized to MP and 375 to conventional bearings. One RCT was excluded from meta-analysis because of high risk of bias. Meta-analysis comparing MP with PS only was possible and found no differences at any time points for any outcome measure, including 2-year follow-up for Oxford Knee Score (MD = 0.35 favoring PS; 95% CI −0.49 to 1.20) and range of motion (MD = 1.58 favoring MP; 95% CI −0.76 to 11.92, *P* = 0.30) and 12 months for Western Ontario Arthritis Index (MD = 4.42 favoring MP; 95% CI −12.04 to 3.20, *P* = 0.09).

**Conclusions:**

There is no difference in clinical outcomes, with contemporary measurement tools, at any time points, between MP and PS. There are insufficient RCTs comparing MP with other bearings.

Primary total knee arthroplasty (TKA) can be performed with various articular bearings. Each influences joint stability, function, and implant survivorship differently, through unique geometries and levels of conformity. Some bearings are fixed to the tibial baseplate: cruciate-retaining (CR), ultracongruent (UC), and posterior-stabilized (PS). Mobile bearings (MBs) are able to move relative to the tibial baseplate. More recently, medial pivot (MP) (or medial stabilized) designs have been developed to replicate some aspects of native knee joint kinematics.^1–3^

There are theoretical kinematic advantages of MP designs. They feature a conforming medial compartment in the sagittal and frontal planes that creates a shallow, “ball and socket” joint. The lateral compartment articulation is less congruent, to permit femoral roll back here and not in the medial compartment in flexion.^4^ The increased medial conformity provides increased sagittal stability and distributes load over a wider surface area.^5^ The adoption of MP designs into practice has been rapid in some regions, comprising for example 9.8% of all TKA performed in Australia.^6^ The incidence of use, complications, survivorship, and modes of failure specific to MP designs remain unclear,^7^ with limited published data.^4^

Previous systematic reviews and meta-analyses have compared MP designs with conventional bearings by including all study designs, irrespective of study quality or risk of bias, and pooling all bearing types together.^8–10^ By including only randomized controlled trials (RCTs) that compare MP designs with specific bearings, better quality evidence is expected. This systematic review and meta-analysis asks (1) in patients receiving primary TKA, do the clinical and patient-reported outcomes and (2) the incidence of complications differ between TKA performed with MP designs and other bearings: CR, UC, PS, or MB?

## Methods

### Search Strategy

This systematic review was performed according to the guidelines of Preferred Reporting Items for Systematic Reviews and Meta-Analyses and protocol registered with PROSPERO (ID:CRD42022300190). A literature search was performed in MEDLINE, EMBASE, PubMed, Web of Science, and Scopus databases using a combination of controlled vocabulary and keywords. The search strategy in MEDLINE (Ovid) :

1. (medial* and (pivot* or stabili* or rotat* or congruent or "ball-and-socket" or "ball-in-socket")).mp.
2. (anteromedial portal or AMP or MRK or SAIPH or GMK or Evolution or Advance).mp.
3. 1 or 2
4. (knee and (replacement* or arthroplast* or TKA or prosthes* or surgery)).mp.
5. (random* control* trial* or random* clinical trial* or RCT*).mp.
6. 3 and 4 and 5
7. Limit 6 to English

The search included studies published in English between January 1, 1986, and March 25, 2022. The search was conducted by two independent investigators (G.S. and W.H.) using the Covidence Systematic Review Software (2021; Veritas Health Innovation). Where there was disagreement over study inclusions, the investigators reviewed the study together until consensus was reached with a further investigator if required (K.V.). The search was supplemented with hand searching conference proceedings and publication reference lists, and experts in the field were contacted to ensure complete capture of the literature.

### Inclusion and Exclusion Criteria

We included RCTs of primary TKA that compared MP designs specifically with CR, UC, PS, or MB and that reported clinical outcomes and patient-reported outcome measures (PROMs) in adult patients aged 18 years and older.^11^ Comparative studies that were not RCT designs, studies that included TKAs performed in patients aged younger than 18 years, studies that included revision TKA, and studies that only compared kinematic assessment of the knee or gait analysis were excluded.

### Data Extraction

Data were extracted by the same two investigators (G.S. and W.H.) into an Excel (2003; Microsoft) spreadsheet, including study methods, participants, interventions, surgical technique, and outcomes. Where data were inadequate or not reported, attempts were made to contact the corresponding authors. The primary outcome measures included all clinical function scores, PROMs, and knee range of motion (ROM). Outcome measures related to kinematic assessment of the knee and gait analysis were not included. The secondary outcome measure was complications, specifically stiffness and aseptic revision. All extracted outcome variables were continuous and the mean differences were used as comparison. The data collected were analyzed using R version 3.6.3 (R Foundation for Statistical Computing) by a single investigator (T.S.).

### Assessment of Risk of Bias

Two review investigators (G.S. and W.H.) independently assessed the risk of bias of the included studies using the Cochrane Risk-of-Bias tool for randomized trials.^12^ Studies with a high risk of bias were excluded from the meta-analysis. No attempt was made to mask the trial reports. Where disagreement existed concerning the assessment, we reached consensus through discussion among all review authors.

### Unit of Analysis Issues

The studies and data included in the final analysis were assessed for potential unit of analysis issues relating to the clustering of patients to the MP intervention or comparator group based on the surgeon or hospital and/or treated with bilateral TKA that were analyzed on a per surgical fixation basis. We expected heterogeneity in follow-up times and planned for pooled analysis of clinical outcomes and PROMs at short and medium intervals after the intervention. Complications were reported at the final follow-up of each study. Not all time points had sufficient data across all outcomes to run a meta-analysis, and only those outcomes and time points for which minimum data were available had meta-analysis performed. Where data were inadequately reported or missing, attempts were made to contact the publishing authors.

### Assessment of Heterogeneity

Heterogeneity (variation in the outcomes between studies) was assessed visually by inspection of forest plots and statistically using *χ*^2^ and I^2^ tests.^13^ A *P* value of <0.1 for *χ*^*2*^ was set to indicate significant heterogeneity. I^*2*^ was interpreted as 0% to 40% might not be important; 30% to 60% may represent moderate heterogeneity; 50% to 90% may represent substantial heterogeneity; 75% to 100% indicated considerable heterogeneity.^14^

### Data Synthesis

Outcome variables that were reported in a comparable manner among studies (criteria set a priori) were included in the meta-analysis. MP designs were compared separately against each bearing type: CR, UC, PS, and MB. Continuous outcomes were compared using a random-effects mean difference meta-analysis regression. A random-effects model was preferred over a fixed-effects approach to control for differences in the treatment effect between studies attributable to differences in study patient populations, settings, and surgeons. Continuous variables were reported as mean ± SD, with the mean weighted for sample size. For all comparisons, *P* < 0.05 was considered significant. All analyses were conducted using R version 3.6.3 (R Foundation for Statistical Computing).

## Results

### Systematic Review

**Figure 1:**
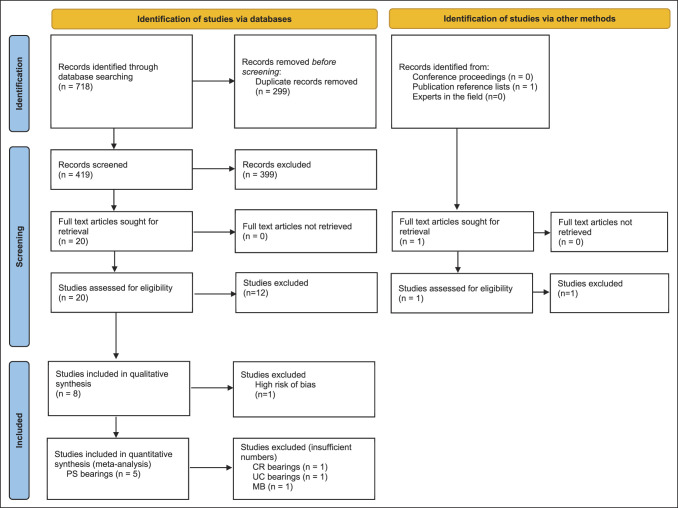
Image showing study flow chart per PRISMA standards. Overall records identified 687 studies with final inclusion of five studies for quantitative analysis. PRISMA = preferred reporting items for systematic reviews and meta-analyses

The search strategy yielded 718 studies with 299 duplicates removed, leaving 419 titles and abstracts for screening. Three hundred eighty studies were deemed irrelevant leaving 20 studies to be assessed for eligibility. Agreement was obtained on eight studies for final inclusion after full-text review (Figure 1). Seven studies were single (assessor) blinded RCTs, and one study was a single (assessor) blinded, three group parallel RCT (Table 1). Five studies compared MP with PS bearings, two with UC, and one with CR and MB. In total, 350 knees were randomized to MP and 375 to conventional bearings (194 PS, 99 MB, 55 UC, and 27 CR) (Table 1). The risk of bias assessment showed overall some concern for risk of bias in five studies and high risk of bias in one study, which was excluded from meta-analysis (Table 2). For the studies with some concern for risk of bias, most issues were due to the randomization process and missing outcomes.

**Table 1:** Summary of Included Studies

Study Author	Journal	Year	Study Country	Study Design	Study Source	Surgeon Number	Funding	Financial Conflict	Inclusion	Exclusion
Batra et al.^15^	KSSTA	2020	India	Single-blinded RCT	Single center	Single surgeon	Nil	Yes	Comparable bilateral KL grade 4 arthritis in ASA 1 or 2 undergoing simultaneous bilateral TKA	History of patellectomy, high tibial osteotomy, BMI >40, those undergoing simultaneous hip and knee arthroplasty
Chang et al.^16^	JOA	2021	The United Kingdom	Single-blinded RCT	Single center	Single surgeon (performed or supervised fellow)	Departmental research funds	Yes	Symptomatic OA requiring primary TKA; fit for surgical intervention; aged 18–80 years; able to give informed consent and comply with postoperative review; sufficient mobility to attend clinics	Unable to tolerate GA; previous infection of knee; revision of UKA; TKA for fracture or previous osteotomy; neurological dysfunction compromising mobility
Dowsey et al.^17^	JOA	2020	Australia	Single-blinded, 3-group parallel RCT	Single center	4	Medacta	Yes	Clinical and radiographic OA, KL grade 24 in patients aged 50-85 years	Revision surgery, surgery for neoplastic disease, inability to perform informed consent because of mental incompetence, active drug or alcohol disorder, limited English, severe deformity, BMI >36, unable to ambulate independently preoperatively, existing TKA in contralateral knee
Edelstein et al.^18^	J Knee Surg	2020	USA	Single blinded RCT	Single center	2	Not reported	None declared	Advanced primary OA who are indicated for TKA and aged 18-85	Secondary OA, history of prior open knee surgery, flexion contracture >20°, valgus deformity >10°
Ishida et al.^19^	KSSTA	2014	Japan	Single-blinded RCT	Single center	Single surgeon	Not reported	Not reported	Varus deformity patients with OA	Valgus deformity, severe bony defects, RA
Kim et al.^20^	CORR	2009	Korea	Single-blinded RCT	Single center	Single surgeon	Not reported	None declared	OA severe enough to warrant TKA after an adequate trial of nonsurgical therapy and the need for bilateral, single-stage TKA	RA, history of septic arthritis
Kulshrestha et al.^21^	CiOS	2020	India	Single-blinded RCT	Single center	Single surgeon	Not reported	None declared	Advanced bilateral OA severe enough for TKA and fit for single-staged bilateral procedures	RA, severe deforming arthritis requiring specialized implants
Nishitani et al.^22^	Knee	2018	Japan	Single-blinded RCT	Not reported	Not reported	Not reported	Yes	OA with varus deformity	Valgus deformity, severe bony defects, RA, a history of revision arthroplasty, bedridden for reasons other than knee surgery

Study Author	Diagnosis for Surgery	Unilateral or Bilateral	MP Prosthesis	Comparator Bearing	Comparison Prosthesis	Sample Size Randomized MP	Sample Size Randomized Comparator	MP Loss to Follow-Up	Comparator Loss to Follow-Up	Outcome Measures	Summary of Results
Batra et al.^15^	OA (87%); RA (13%)	Bilateral, single stage	ADVANCE (MicroPort)	PS	Genesis II (Smith and Nephew)	53	53	0	0	KSS (satisfaction); KSS (expectation) OKS; kinematic assessment at 6 months, ROM	Benefit favoring MP at 3 months - 4 years for KSS satisfaction; from 6 months - 4 years for KSS expectation; no difference in OKS or ROM; high patella tendon angle in MP throughout ROM
Chang et al.^16^	OA	Unilateral	SAIPH (MatOrtho)	PS	Triathlon (Stryker)	44	45	6	5	ROM; WOMAC; OKS; AKSS; SF-36	No difference at 1 or 2 years for ROM, WOMAC, OKS, AKSS
Dowsey et al.^17^	OA	Unilateral	GMK sphere (medacta)	PS & CR	GMK-PS or GMK-CR (Medacta)	29	26 PS & 27 CR	2	3 PS & 2 CR	OKS; WOMAC; KSS; 6MWT; TUG; VR 12	No difference in OKS at 6 months; at 6 months MP favored over CR and PS for satisfaction; at 12 months MP and PS benefits over CR for WOMAC
Edelstein et al.^18^	OA	Unilateral	GMK sphere (medacta)	PS	GMK-PS (Medacta)	30	30	5	5	OKS; VR 12; IKDC; PROMIS; KSS; FJS; satisfaction questions; stability testing using KT-1000 arthrometer; ROM; TUG	MP greater sagittal stability at 30° but not 90°, no difference in ROM, PROMIS, OKS, KSS, FJF, VR12
Ishida et al.^19^	OA	Unilateral	ADVANCE MP (MicroPort)	UC	ADVANCE DH (MicroPort)	20	20	0	0	KSS, KSFS, ROM, UCLA activity level	No difference at 2-year and greater follow-up for ROM, KSS, KSFS and UCLA activity score
Kim et al.^20^	OA	Bilateral, single stage	Medial pivot (wright medical)	MB	PFC Sigma (DePuy)	99	99	7	7	ROM; KSS; KSFS; HSS knee score; patient satisfaction VAS	Benefit favoring MB for ROM, KSS, HSS scores
Kulshrestha et al.^21^	OA	Bilateral, single stage	ADVANCE (MicroPort)	PS	NexGen Legacy (Zimmer)	40	40	4	3	ROM; KSS; DOPS; FJS; EQ5D	Benefit for PS for ROM, benefit for TUG for MP, no difference in new KSS and FJS
Nishitani et al.^22^	OA	Unilateral and bilateral	Bi-surface MP (Kyocera)	UC	Bisurface SD (Kyocera)	35	35	2	3	ROM, 1989 and 2011 KSS, KSFS	No difference in ROM, KSS, KSFS at 2 years

6DOF = 6 Degrees of Freedom, 6MWT = 6-minute walk test, AKSS = American Knee Society Score, ASA = American Society of Anesthesiologists, BMI = body mass index, CiOS = Clinics in Orthopaedic Surgery, CORR = clinical orthopaedics & related research, CR = cruciate retaining, DOPS = delaware osteoarthritis profile score, EQ5D = EuqoQol five Dimension, FJF = forgotten joint score, HSS = Hospital For Special Surgery, HTO = high tibial osteotomy, IKDC = international knee documentation score, J Knee Surg = Journal of Knee Surgery, JOA = Journal of Arthroplasty, KL = Kellgren-Lawrence, KSFS = knee society functional score, KSS = Knee Society Score, KSSTA = Knee Surgery Sports Traumatology Arthroscopy, Los Angeles, MB = mobile bearing, MP = medial pivot, OA = osteoarthritis, OKS = Oxford knee score, PROMIS = patient-reported outcomes measurement information system, PS = posterior stabilized, RA = rheumatoid arthritis, ROM = knee range of motion, SF-36 = 36-Item Short Form Health Survey, TKFQ = total knee function questionnaire, TUG = timed up and go test, UC = ultracongruent, UCLA = University of California, UKA = unicompartmental knee replacement, VR 12 = Veterans RAND 12 Item Health Survey, WOMAC = Western Ontario and McMaster Universities Arthritis Index

**Table 2:** Cochrane Risk of Bias Assessment for Randomized Trials

Study author	Journal	Year	Assessor	RoB2 Domains					Overall Risk of Bias
				Randomization Process	Deviations From the Intended interventions	Missing Outcomes	Measurement of Outcomes	Selection of Reported Results	
Batra et al.^15^	KSSTA	2020	GS	Low	Low	Low	Low	Low	Low
			WH	Low	Low	Low	Low	Low	Low
Chang et al.^16^	JOA	2021	GS	Low	Low	Some concern	Low	Low	Some concern
			WH	Low	Low	Some concern	Low	Low	Some concern
Dowsey et al.^17^	JOA	2020	GS	Some concern	Low	Low	Low	Low	Some concern
			WH	Some concern	Low	Low	Low	Low	Some concern
Edelstein et al.^18^	J knee surg	2020	GS	Some concern	Low	Some concern	Low	Some concern	Some concern
			WH	Some concern	Low	Some concern	Low	Some concern	Some concern
Ishida et al.^19^	KSSTA	2014	GS	Some concern	Low	Low	Low	Low	Some concern
			WH	Some concern	Low	Low	Low	Low	Some concern
Kim et al.^20^	CORR	2009	GS	Low		Some concern	Low	Low	Some concern
			WH	Low	Low	Some concern	Low	Low	Some concern
Kulshrestha et al.^21^	CiOS	2020	GS	Low	Low	Low	Low	Low	Low
			WH	Low	Low	Low	Low	Low	Low
Nishitani et al.^22^	Knee	2018	GS	Low	Low	Low	High	Low	High
			WH	Low	Low	Low	High	Low	High

CiOS = Clinics in Orthopaedic Surgery, CORR= Clinical Orthopaedics & Related Research, J Knee Surg = Journal of Knee Surgery, JOA= Journal of Arthroplasty, KSSTA= Knee Surgery, Sports Traumatology, Arthroscopy, RoB2 = Risk of Bias 2

Patient characteristics for the studies included are shown in Table 3. The raw data for the primary outcome measures are presented in Tables 4 to 6 and for the secondary outcome measures in Table 7.

**Table 3:** Patient Characteristics of Included Studies

Study Author	Journal	Year	Comparator Bearing	Sample Size Randomized MP	Sample Size Randomized Comparator	MP Loss to Follow-Up	Comparator Loss to Follow-Up	Unilateral or Bilateral Surgery
Batra et al.^15^	KSSTA	2020	PS	53	53	0	0	Bilateral, single stage
Chang et al.^16^	JOA	2021	PS	44	45	6	5	Unilateral
Dowsey et al.^17^	JOA	2020	PS	29	26	2	3	Unilateral
Edelstein et al.^18^	J knee surg	2020	PS	30	30	5	5	Unilateral
Kulshrestha et al.^21^	CiOS	2020	PS	40	40	4	3	Bilateral, single stage

Study Author	Mean Age MP (SD)	Mean Age Comparator (SD)	Female % MP	Female % Comparator	Mean BMI MP (SD)	Mean BMI Comparator (SD)	Baseline Differences	Follow-Up
Batra et al.^15^	61.7 (6.9)	61.7 (6.9)	67	67	28.3 (3.4)	28.3 (3.4)	Matched for all preoperative parameters	3 months, 6 months, 4 years
Chang et al.^16^	68.4 (5.7)	69.1 (5.4)	69	62	29.2 (3.8)	28.8 (3.4)	Matched for all preoperative parameters	1 year, 2 years
Dowsey et al.^17^	66.0 (6.8)	65.7 (7.7)	52	42	Not reported	Not reported	Higher BMI and higher proportion of people with multiple comorbidities in MP; older age in CR; balanced randomization across surgeons	6 weeks, 6 months, 1 year
Edelstein et al.^18^	67.0 (8)	64.0 (7)	72	60	32.8 (5.8)	34.2 (5.8)	Matched for all preoperative parameters	6 weeks, 3 months, 6 months, 1 year, 2 years
Kulshrestha et al.^21^	63.8 (6.8)	66.0 (6.7)	73	58	27.3 (5.1)	26.6 (4.3)	Matched for all preoperative parameters	6 weeks, 3 months, 6 months, 1 year, 2 years

CiOS = Clinics in Orthopaedic Surgery, CORR = Clinical Orthopaedics & Related Research, J Knee Surg = Journal of Knee Surgery, JOA = Journal of Arthroplasty, KSSTA = Knee Surgery, Sports Traumatology, Arthroscopy

**Table 4:** Raw Data for the Oxford Knee Score

Study Author	Journal	Year	Comparator Bearing	Sample Size Randomized MP	Sample Size Randomized Comparator	MP Loss to Follow-Up	Study Author	OKS MP Preoperative
Batra et al.^15^	KSSTA	2020	PS	53	53	0	Batra et al.	9.2 (2.8)
Chang et al.^16^	JOA	2021	PS	44	45	6	Chang et al.	21.9 (4.8)
Dowsey et al.^17^	JOA	2020	PS	29	26	2	Dowsey et al.	17.8 (7.5)
Edelstein et al.^18^	J Knee Surg	2020	PS	30	30	5	Edelstein et al.	16.3 (7.7)
Kulshrestha et al.^21^	CiOS	2020	PS	40	40	4	Kulshrestha et al.	N/A

Study Author	OKS PS Preoperative	OKS MP 3 mo	OKS PS 3 mo	OKS MP 6 mo	OKS PS 6 mo	OKS MP 1 yr	OKS PS 1 yr	OKS MP 2 yr (or greater)	OKS PS 2 yr (or greater)
Batra et al.^15^	9.3 (3.0)	39.4 (2.9)	39.3 (2.9)	41.3 (2.6)	41.3 (2.8)	Not reported	Not reported	44.3 (2.2)	44.0 (2.3)
Chang et al.^16^	22.4 (5.1)	Not reported	Not reported	Not reported	Not reported	41.9 (9.7)	41.1 (7.5)	42.7 (8.1)	42.3 (6.7)
Dowsey et al.^17^	16.2 (6.0)	Not reported	Not reported	33.4 (9.3)	34.5 (9.8)	32.4 (13.2)	33.8 (13.4)	N/A	N/A
Edelstein et al.^18^	19.9 (9.4)	32.0 (11.4)	32.7 (9.5)	33.7 (8.9)	36.4 (10.6)	39.2 (10.0)	35.8 (11.3)	40.4 (8.6)	38.3 (11.8)
Kulshrestha et al.^21^	N/A	N/A	N/A	N/A	N/A	N/A	N/A	N/A	N/A

CiOS = Clinics in Orthopaedic Surgery, CORR = Clinical Orthopaedics & Related Research, J Knee Surg = Journal of Knee Surgery, JOA = Journal of Arthroplasty; KSSTA = Knee Surgery, Sports Traumatology, Arthroscopy

**Table 5:** Raw Data for WOMAC

Study Author	Journal	Year	Comparator Bearing	Sample Size Randomized MP	Sample Size Randomized Comparator	MP Loss to Follow-Up	Comparator Loss to Follow-Up
Batra et al.^15^	KSSTA	2020	PS	53	53	0	0
Chang et al.^16^	JOA	2021	PS	44	45	6	5
Dowsey et al.^17^	JOA	2020	PS	29	26	2	3
Edelstein et al.^18^	J Knee Surg	2020	PS	30	30	5	5
Kulshrestha et al.^21^	CiOS	2020	PS	40	40	4	3

Study Author	WOMAC MP Preoperative	WOMAC PS Preoperative	WOMAC MP 6 mo	WOMAC PS 6 mo	WOMAC MP 1 yr	WOMAC PS 1 yr	WOMAC MP 2 yr	WOMAC PS 2 yr
Batra et al.^15^	N/A	N/A	N/A	N/A	N/A	N/A	N/A	N/A
Chang et al.^16^	60.5 (19.9)	58.1 (18.7)	Not reported	Not reported	27.3 (23.7)	32.3 (16.8)	26.8 (19.8)	22.0 (12.0)
Dowsey et al.^17^	57.0 (20.2)	59.6 (14.4)	22.6 (14.3)	29.9 (20.3)	19.2 (14.8)	23.0 (18.1)	N/A	N/A
Edelstein et al.^18^	N/A	N/A	N/A	N/A	N/A	N/A	N/A	N/A
Kulshrestha et al.^21^	N/A	N/A	N/A	N/A	N/A	N/A	N/A	N/A

CiOS = Clinics in Orthopaedic Surgery, CORR = Clinical Orthopaedics & Related Research, J Knee Surg = Journal of Knee Surgery, JOA = Journal of Arthroplasty; KSSTA = Knee Surgery, Sports Traumatology, Arthroscopy, WOMAC = Western Ontario Arthritis Index

**Table 6:** Raw Data for ROM

Study Author	Journal	Year	Comparator Bearing	Sample Size Randomized MP	Sample Size Randomized Comparator	MP Loss to Follow-Up	Preoperative ROM MP
Batra et al.^15^	KSSTA	2020	PS	53	53	0	96 (13.6)
Chang et al.^16^	JOA	2021	PS	44	45	6	97.4 (7.2)
Dowsey et al.^17^	JOA	2020	PS	29	26	2	N/A
Edelstein et al.^18^	J Knee Surg	2020	PS	30	30	5	Not reported
Kulshrestha et al.^21^	CiOS	2020	PS	40	40	4	113.9 (7.7)

Study Author	Preoperative ROM PS	ROM MP 1 yr	ROM PS 1 yr	ROM MP 2 yr (or greater)	ROM PS 2 yr (or greater)	ROM MP Final Follow-Up	ROM PS Final Follow-Up	Notes
Batra et al.^15^	99 (11.5)	Not reported	Not reported	118 (8.6)	116 (9.3)	118 (8.6)	116 (9.3)	
Chang et al.^16^	94.8 (4.9)	114.6 (16.3)	111.3 (17.8)	114.9 (15.5)	114.9 (16.4)	114.9 (15.5)	114.9 (16.4)	
Dowsey et al.^17^	N/A	N/A	N/A	N/A	N/A	N/A	N/A	
Edelstein et al.^18^	Not reported	111.2 (10.4)	114.7 (10.7)	N/A	N/A	111.2 (10.4)	114.7 (10.7)	1 year data used as final follow-up
Kulshrestha et al.^21^	108.8 (16.4)	Not reported	Not reported	Not reported	Not reported	Not reported	Not reported	

CiOS = Clinics in Orthopaedic Surgery; CORR = Clinical Orthopaedics & Related Research; J Knee Surg = Journal of Knee Surgery; JOA = Journal of Arthroplasty; KSSTA = Knee Surgery, Sports Traumatology, Arthroscopy, ROM = range of motion

**Table 7:** Raw Data for Complications

Study Author	Journal	Year	Comparator Bearing	Sample Size Randomized MP	Sample Size Randomized Comparator	MP Loss to Follow-Up	Stiffness MP	Stiffness PS	Aseptic Revisions MP	Aseptic Revisions PS
Batra et al.^15^	KSSTA	2020	PS	53	53	0	0	0	0	0
Chang et al.^16^	JOA	2021	PS	44	45	6	1	3	1	0
Dowsey et al.^17^	JOA	2020	PS	29	26	2	2	1	0	0
Edelstein et al.^18^	J Knee Surg	2020	PS	30	30	5	Not reported	Not reported	Not reported	Not reported
Kulshrestha et al.^21^	CiOS	2020	PS	40	40	4	0	1	0	0

CiOS = Clinics in Orthopaedic Surgery, CORR = Clinical Orthopaedics & Related Research, J Knee Surg = Journal of Knee Surgery, JOA = Journal of Arthroplasty, KSSTA = Knee Surgery, Sports Traumatology, Arthroscopy

### Meta-Analysis

No meta-analysis was possible of comparison of MP designs with CR, UC, or MB because too few RCT's had been performed. Meta-analysis of five studies that compared MP (196 knees) with PS bearings (194 knees) was performed. There were 15 different clinical outcome measures or PROMs used across the five studies. Meta-analysis was only possible on three of these outcome measures: Oxford Knee Score (OKS), Western Ontario Arthritis Index (WOMAC), and ROM, as the other outcome measures were not used in a sufficient number of studies to allow for data pooling and comparison.

#### Oxford Knee Score

**Figure 2:**
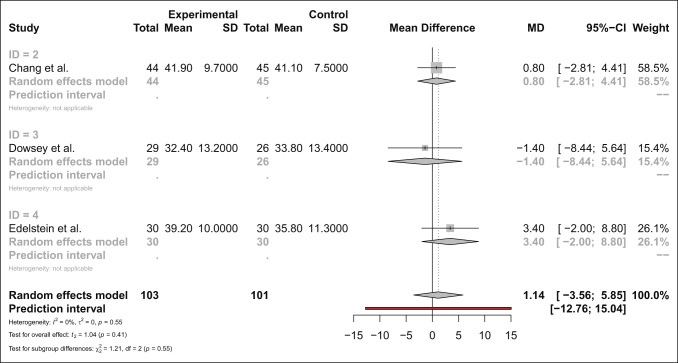
Forest plot comparing 2-year or greater follow-up of Oxford Knee Scores in MP designs and PS bearings. MP = medial pivot, PS = posterior-stabilized

There was no notable difference in OKS between MP and PS bearings at any time point. Four studies compared preoperative OKS for 156 MP and 154 PS bearing groups and found no difference (mean difference 0.26 favoring MP [95% CI −2.12, 1.60, *P* = 0.69]). There was no difference at 3 months, 6 months, and 12 months, and three studies compared 2-year or greater OKS for 127 MP and 128 PS bearings and found no difference (mean difference 0.35 favoring PS [95% CI −0.49 to 1.20, *P* = 0.22]) (Figure 2). The interaction test for subgroup difference did not suggest that the relationship was likely to be important (*χ*^2^ = 0.45, *P* = 0.80).

#### Western Ontario Arthritis Index

**Figure 3:**
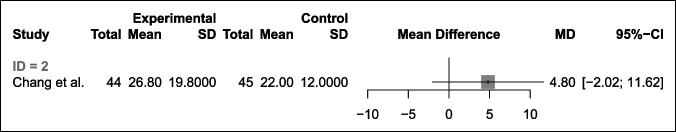
Forest plot comparing 12-month follow-up of Western Ontario Arthritis Index in MP designs and PS bearings. MP = medial pivot, PS = posterior-stabilized

There was no notable difference in WOMAC scores between MP and PS bearings at any time point. Two studies compared preoperative WOMAC for 73 MP and 74 PS bearings and found no difference (mean difference 0.24 favoring MP [95% CI −31.23, 31.71, *P* = 0.94]). Two studies compared WOMAC scores at 12-months for 73 MP and 71 PS bearings and found no difference (mean difference 4.42 favoring MP [95% CI −12.04, 3.20, *P* = 0.09]) (Figure 3). The interaction test for subgroup difference did not suggest that the relationship was likely to be important (*χ*^2^ = 0.04, *P* = 0.85).

#### Range of motion

**Figure 4:**
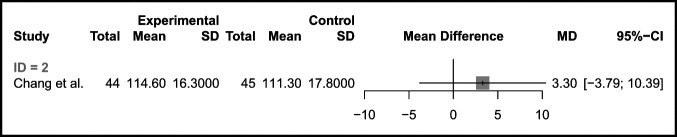
Forest plot comparing 2-year or greater follow-up of ROM in MP designs and PS bearings. MP = medial pivot, PS = posterior-stabilized, ROM = range of motion

There was no notable difference in ROM between MP and PS bearings at any time point. Three studies compared preoperative ROM for 137 MP and 138 PS bearings and found no difference (mean difference 1.56 favoring PS (95% CI −8.10, 11.21, *P* = 0.56). Two studies compared the ROM at 2 years or greater follow-up for 97 MP and 98 PS bearing and found no difference (mean difference 1.58 favoring MP [95% CI −0.76, 11.92, *P* = 0.30]) (Figure 4). The interaction test for subgroup difference did not suggest that the relationship was likely to be important (*χ*^2^ = 0.28, *P* = 0.60).

#### Other Complications

**Figure 5:**
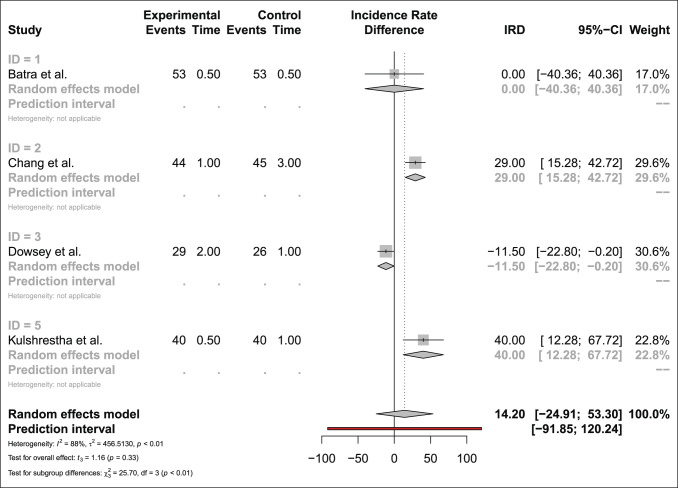
Forest plot comparing stiffness-related complications in MP designs and PS bearings. MP = medial pivot, PS = posterior-stabilized

Four studies compared the risk of stiffness-related complications for 166 MP and 164 PS bearings. The estimated risk difference showed no significant difference between MP and PS bearings (estimated incidence rate difference 14.20 favoring MP [less stiffness] 95% CI −24.91, 53.30, *P* = 0.33) (Figure 5).

**Figure 6:**
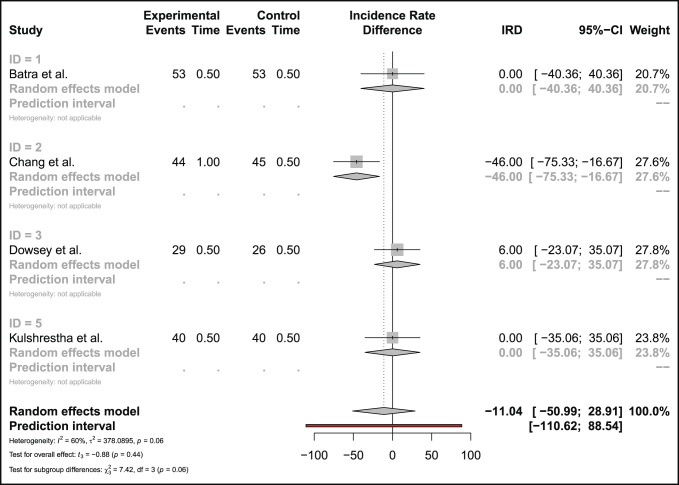
Forest plot comparing risk of aseptic revision in MP designs and PS bearings. MP = medial pivot, PS = posterior-stabilized

Four studies compared the risk of aseptic revision for 166 MP and 164 PS bearings. The estimated risk difference showed no significant difference between MP and PS bearings (estimated incidence rate difference 11.04 favoring PS [less aseptic revisions] 95% CI −50.99, 28.919, *P* = 0.44) (Figure 6).

## Discussion

The difference in clinical outcomes, PROMs, and complications for primary TKA performed with MP designs compared with conventional bearings is not known, and there is increased use of MP designs. This systematic review and meta-analysis of RCTs concluded that, to date, no differences have been documented in the short-term clinical outcomes, PROMs, or complications between MP and PS bearings in TKA, with conventional measurement instruments, at any time point after surgery. Additional RCTs will be required to confirm or refute these findings. There are insufficient RCTs that compare MP designs with other bearings: CR, UC, or MB. Accordingly, the differences between MP designs and these bearings have not been determined.

To our knowledge, this is the first systematic review of RCTs that compared MP designs with conventional bearings, although meta-analysis was limited to comparison with PS bearings. Other systematic reviews and meta-analyses are limited by pooling of all study designs (retrospective and prospective), inclusion of study designs with a high risk-of-bias, pooling of all conventional bearing types into one group, and heterogeneity of included patients,^8–10^ such as RCTs including both primary and revision procedures.^23^ Notwithstanding, those reviews support our findings of no clear difference in clinical outcomes between MP and PS bearings.^8–10^ The number of outcome measures that could be included in the meta-analysis was limited by inconsistency of outcome measures between studies, variation in the time of outcome measure reporting, and other study limitations with the reporting of results. Standardization of data reporting would improve the RCTs conducted and the ability to perform meta-analysis.^24^ Meta-analysis was accordingly limited to three clinical outcome measures and PROMs. Although validated and widely used, the relatively crude outcome measures included in our meta-analysis evaluate general function of a TKA. It is possible that differences may exist between MP and other TKA designs when alternate outcome measures are used, including those with lower ceiling effects or more demanding performance tests, which might identify small but notable differences between arthroplasty designs.

Some of the hypothesized kinematic improvements of MP designs have been realized in gait analysis, but postoperative kinematics for MP (and CR and PS) bearings still do not match a native, nonarthritic joint^3^ nor is benefit always seen.^25^ Along with the hypothetical benefits, there are hypothetical disadvantages too. The increased conformity may lead to component impingement, which may limit femoral roll back and flexion in some patients.^26^ Kinematic conflict can result when the articular geometry does not match the soft-tissue kinematics.^27^ Retaining the PCL for example with the increased MP conformity can cause this, and two included studies recessed or selectively sacrificed the PCL.^20,21^ Differences in surgical techniques may influence outcomes.

The successful TKA achieves both good clinical function and implant durability. The equivocal clinical results of MP designs when compared with PS bearings may support MP uptake. PS bearings have been the most common bearing in the United States,^28^ where their use is currently declining, as in other countries.^6,29,30^ One explanation for the decline of PS bearings is the increased long-term risk of revision with PS compared with CR bearings.^6,31^ However, MP and PS bearings may have the same long-term revision risk, with CR bearings having a lower long-term risk.^6,7^ This makes future RCTs comparing MP and CR bearings important, given an apparent difference in survivorship. CR bearings are the most common type of bearing used in primary TKA in Australia,^6^ New Zealand,^29^ the United Kingdom^30^ and now in the United States.^28^ The current difference in long-term revision rates of MP designs may change with follow-up of newer MP designs. None of the newest designs have greater than five years documented follow-up.^6^ Long-term follow-up might be influenced by polyethylene quality, independent of the bearing design. No MP design reported in the Australian Registry is manufactured with highly cross-linked polyethylene (XLPE), a material known to decrease component loosening and revisions.^6,32^ How the increased conformity of MP designs affect polyethylene wear is unknown. There are also variations in survivorship between individual MP designs,^7,33^ which makes grouping MP designs vexed. An early MP design, for example, ADVANCE (MicroPort, Shanghai, China), experienced high revision rates and is excluded from comparisons in the Australian Registry.^6^ There are also concerns for the durability of some modern MP designs. Roentgen stereophotogrammetric analysis of the GMK Sphere (Medacta, Castel San Pietro, Switzerland) found comparatively high early tibial tray motion, usually associated with aseptic loosening.^34^

There are limitations to this study. First, there are few RCTs which directly compare MP designs with conventional bearings. Other limitations relate to the inclusion and exclusion criteria of the included studies. Of the studies included in the systematic review, three solely included bilateral single-stage TKA,^15,20,21^ and one study included both unilateral and bilateral TKA.^22^ Two of these studies were included in the meta-analysis,^15,21^ and it is unclear whether these patient populations represent patients in general. Furthermore, both the studies that included bilateral single-stage TKA recruited patients from India where the severity of arthritis, patient expectations, and postoperative rehabilitation may be distinct. Batra et al.^15^ from India studied only Grade 4 Kellgren-Lawrence arthritic changes. Dowsey et al^17^ (by comparison, working in Australia) included patients with Grade 2 to 4 changes. Other studies failed to quantify the preoperative status of patients. Batra et al^15^ were alone in including patients with rheumatoid arthritis (13% of cases). Limitations and generalizability also relate to preoperative limb alignment. Two studies restricted inclusion to preoperative varus alignment and excluded valgus^19,22^ while another excluded valgus >10°.^18^ Of note, all studies but two^15,21^ reported a goal of neutral (mechanical) alignment, despite recent interest in alternative TKA alignments.

## Conclusions

This systematic review and meta-analysis of RCTs provide evidence that there are no, as yet identifiable, short-term differences between MP and PS bearings for clinical outcomes, PROMs, or complications in primary TKA, at any time point. There are insufficient RCTs to compare MP with other conventional bearings, and the clinical differences are unexplored and unknown. Additional RCTs will be required that use consistent outcome measures, including those with lower ceiling effects, with standardization of data reporting to define advantages of one TKA design over another. Continued monitoring of revision rates by registries is mandatory.
